# P-433. Identifying Risk Factors for Single versus Recurrent Community Associated Staphylococcus aureus Skin and Soft Tissue Infections among Pediatric Patients in the Atlanta area (2002-2019)

**DOI:** 10.1093/ofid/ofaf695.649

**Published:** 2026-01-11

**Authors:** Declan Quinn, Shifan Yan, Traci Leong, Samuel Owusu, Daria Anderson, Xiting Lin, Peter T Baltrus, Lilly Immergluck

**Affiliations:** University of Chicago, Elmhurst, Illinois; Emory University, Atlanta, Georgia; Emory University, Atlanta, Georgia; Morehouse School of Medicine, Atlanta, Georgia; Morehouse School of Medicine, Atlanta, Georgia; Morehouse School of Medicine, Atlanta, Georgia; Morehouse School of Medicine, Atlanta, Georgia; University of Chicago, Elmhurst, Illinois

## Abstract

**Background:**

*Staphylococcus aureus* is a gram-positive bacterium that is estimated to colonize up to 30% of the human population. *S. aureus* is also the most common pathogen isolated from skin and soft tissue infections (SSTI). There are numerous strains of *S. aureus*, including methicillin resistant *Staphylococcus aureus* (MRSA) and methicillin susceptible *Staphylococcus aureus* (MSSA). Community-acquired MRSA (CA-MRSA) has been identified to be highly aggressive in causing SSTI. African Americans and children have been identified as the two groups disproportionately affected by CA-MRSA infections. Recurrence of S. aureus SSTI can be seen in 1 in 6 individuals.

**Methods:**

A retrospective study that uses electronic health records from a network of pediatric emergency departments in the Atlanta area from 2002-2019. The study population included pediatric patients (< 19) who were identified to have positive *S. aureus* SSTI. Individual patient level variables associated with sociodemographic characteristics were collected including race, ethnicity, gender, and health insurance. Analyses were performed and stratified by those with single versus recurrent SSTI. Chi-squared tests were used to detect differences between patients with recurrent SSTI from non-recurrent SSTI. We will be applying multi-level analysis using individual patient level data from EHR linked to area level data based on patients’ place of residence at the time of infection and linked to U.S. Census data.

**Figure 2:**
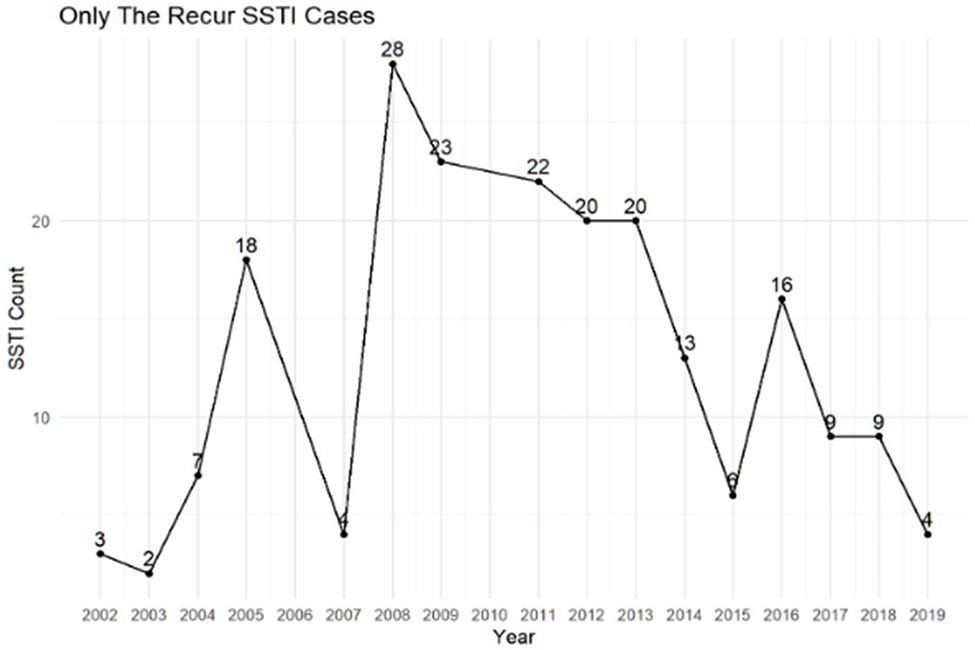
Number of recurrent SSTI patients by year

**Results:**

There was a total of 11,489 SSTI from 11,261 patients between the years 2002-2019. 11,057 of these patients were single episode SSTI. The results showed that patients on public health insurance are at significantly more risk for SSTI than those on private health insurance. Patients on public insurance accounted for over 55% of the study population. The results also show that MRSA caused the majority of infections, both recurrent and non-recurrent, infecting over 60% patients.

**Conclusion:**

There are numerous sociodemographic characteristics that affect one’s susceptibility to *S. aureus* SSTI, including age, gender, and health insurance. Our findings show that SSTI are more commonly associated with MRSA over MSSA and recurrence risk was lower than what has been previously reported.

**Disclosures:**

Lilly Immergluck, MD, MS, American Academy of Pediatrics: Board Member|Department of Energy: Grant/Research Support|moderna: Grant/Research Support|NIH: Grant/Research Support|Pfizer: Grant/Research Support|Sanofi: Grant/Research Support

**Table 1:**
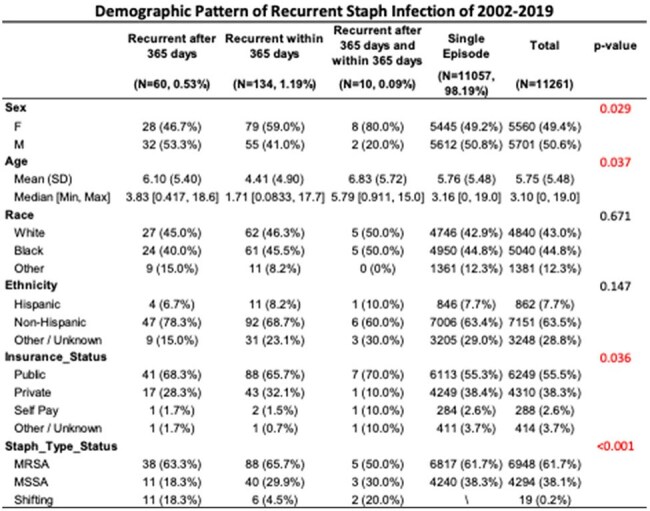
Demographic data of SSTI patient population.

**Figure 1:**
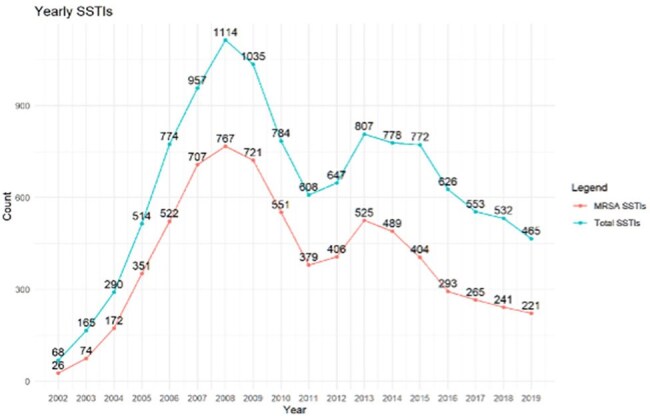
MRSA SSTI compared to total S. aureus SSTI by year.

